# Use of an elevated platform with perforated surface and manure belt by fast-growing broilers on commercial farms

**DOI:** 10.1016/j.psj.2023.103243

**Published:** 2023-10-26

**Authors:** F. May, B. Spindler, J. Müsse, K. Skiba, N. Kemper, J. Stracke

**Affiliations:** ⁎ Institute for Animal Hygiene, Animal Welfare and Farm Animal Behavior, University of Veterinary Medicine Hannover (Foundation), 30173 Hannover, Germany; † Department 3.7, Research Livestock, Chamber of Agriculture Lower Saxony, 26121 Oldenburg, Germany; ‡ Institute of Animal Science, Ethology, University of Bonn, 53115 Bonn, Germany

**Keywords:** broiler, elevated platform, perforated surface, behavior, environmental enrichment

## Abstract

Like other members of the species *Gallus gallus*, fast-growing broilers are motivated to perch. However, broilers in the European Union are kept in unstructured barns, with no opportunity to sit elevated and rest undisturbed. A possible solution to this problem is elevated platforms. The aim of this study was to evaluate the use of an elevated platform with perforated surface and manure belt by fast-growing broilers. On 2 commercial farms, an elevated platform was installed in 1 barn per farm. Approximately 35,000 Ross 308 broilers were housed in each barn for 3 fattening periods. On 1 d per wk, the number of broilers per m² on the platform and the ramp was determined every 30 min from video recordings. Besides, focal animals were observed to analyze their behavior on the platform at different ages and during light and dark periods. Broilers used the elevated platform and the ramp from the first week until the end of the fattening period (platform: 9.92 broilers per m², ramp: 6.47 broilers per m²), with a peak in the fourth week of life (platform: 13.00 broilers per m²). In wk 2, 4, and 5, platform use was higher during the light period than during the dark period. Broilers stayed longer on the platform in the dark period (dark: 01:54:23 [hh:mm:ss], light: 00:19:54 [hh:mm:ss]). In every phase of the fattening period, broilers on the platform were inactive to a high proportion (on average 80.60%). This indicates that broilers used the platform also for resting behavior. Thus, the elevated platform with perforated surface and manure belt is a suitable option to structure broiler barns. It allows the broilers to sit elevated and provides additional space. Factors such as a shallow ramp incline of 20°, wide ramps, and appropriate material used for the surface and ramps may have contributed to its high use by broilers of all ages. Further research is needed to evaluate the design of platforms that allow broilers to rest undisturbed.

## INTRODUCTION

In recent years, the world market for poultry meat has grown steadily. Especially the production of chicken meat continues to increase (Beck, 2022). In the European Union (EU) alone, approximately 6 billion broiler chickens are raised each year, representing 13.3 million tons of poultry meat (EFSA AHAW Panel, 2023). At the same time, consumers and Nongovernmental Organizations, such as the 30 European organizations that signed the European Broiler Commitment, are demanding higher standards for broiler production (BetterChickenCommitment.com, 2023). One of the points of criticisms is the barren environment in broiler barns in the EU, which are equipped only with feed and water lines. In this unstructured environment, possibilities for the birds to express species-specific behavior, such as elevated resting, are limited. In Germany, some farm managers are motivated to integrate elevated platforms into their barns to provide for animal behavioral needs and to meet public demands. A variety of different platform designs can be found (perforated or closed surface, with ramps, suspended or fixed on the barn floor). For commercial farms, platform design should be practical for daily operations in the barn. Further, the design has to be adapted to the limited physical abilities of fast-growing broiler chickens (Malchow et al., 2019).

The domestic chicken (*Gallus gallus domesticus*) is highly motivated to roost on elevated structures at nighttime (McBride et al., 1969; Olsson and Keeling, 2000; Newberry et al., 2001). Chicks are brooded by the hen on the ground until they are developed enough to perch (Newberry et al., 2001). Under feral conditions, the broody hen will encourage her chicks to visit lower branches to rest at night when they are about 6-wk old (McBride et al., 1969). In commercial husbandry, layer chicks start to perch at night at about 3 wk of age, although they are reared without a broody hen (Heikkilä et al., 2006; Riber et al., 2007). Unlike laying hens, fast-growing broiler chickens are limited in their mobility and their behavior is altered by high body weights and associated body imbalances (Weeks et al., 2000; Bessei, 2006; Bailie et al., 2018; Tickle et al., 2018). Nevertheless, they are motivated to use elevated structures when they are adapted to their physical abilities (Norring et al., 2016; Bailie et al., 2018; Malchow et al., 2018; Bach et al., 2019; Malchow et al., 2019; Baxter et al., 2020; Spieß et al., 2022). Previous studies showed that perches are rarely used by broilers, presumably because they have difficulties maintaining balance (Norring et al., 2016; Malchow et al., 2018). Instead, elevated platforms have proven to be a good alternative to perches regarding daytime use (Norring et al., 2016; Bailie et al., 2018; Bach et al., 2019; Malchow et al., 2019; Baxter et al., 2020). Equipped with ramps with a shallow angle of ascent from 14.5° to 35°, they are used by chicks as well as by broilers at slaughter age (Norring et al., 2016; Bach et al., 2019; Malchow et al., 2019; Baxter et al., 2020).

The results of studies evaluating nighttime roosting of fast-growing broiler chickens on elevated platforms are currently inconclusive. A higher use in the dark periods from the third week of life onward was observed by Schomburg et al. (2022). Norring et al. (2016) and Malchow et al. (2019) found higher use of elevated platforms during the light phase and therefore predict that platforms are not primarily used for nighttime roosting. However, there is evidence that broilers rest more undisturbed on elevated platforms during the day and night than on the barn floor where they are often jostled and disturbed by active broilers (Forslind et al., 2021). Particularly at high stocking densities, disturbances of resting broilers increase (Buijs et al., 2010; Spindler and Hartung, 2010; Ventura et al., 2012). Therefore, elevated platforms can contribute to undisturbed rest, by providing additional space, if the area under the platform is accessible for the broilers. The importance of undisturbed rest for broiler chickens was outlined by Schwean-Lardner et al. (2014), Yngvesson et al. (2018), and Forslind et al. (2021), as it is essential for vital functions and animal welfare. Platforms with a perforated surface can even have a positive effect on footpad and hock health (Adler et al., 2020; Tahamtani et al., 2020; Sonnabend et al., 2022) due to the reduced time the broilers spent in soiled and humid litter. In a study with an elevated perforated flooring system that covered 50% of the barn floor and was 15 cm high, more broilers were observed on the elevated floor than in a similarly littered control area (May et al., 2022). This result suggests that the elevated perforated floor was attractive to the broilers. However, a study by Adler et al. (2021) showed that the accumulation of manure under the perforated floor resulted in higher ammonia emissions than in a littered control barn. A possible solution to this problem could be to install a manure belt under the perforated floor to transport manure out of the barn during the fattening period.

Therefore, this on-farm study aimed to evaluate the use of an elevated platform with a perforated floor and manure belt by fast-growing broilers. In contrast to previous studies, the elevated platform was equipped with a water line and an additional light source. The study assessed under commercial conditions how broilers use the platform and ramps in different ages and whether the broilers use the platform to sit elevated during the dark period. Further, the duration of different behavioral patterns and the total time broilers stayed on the platform were analyzed. We predicted that age and lighting phase would influence behavior, with a longer duration of sitting at the end of the fattening period and during dark periods.

## MATERIALS AND METHODS

### Birds and Housing

**Figure 1:**
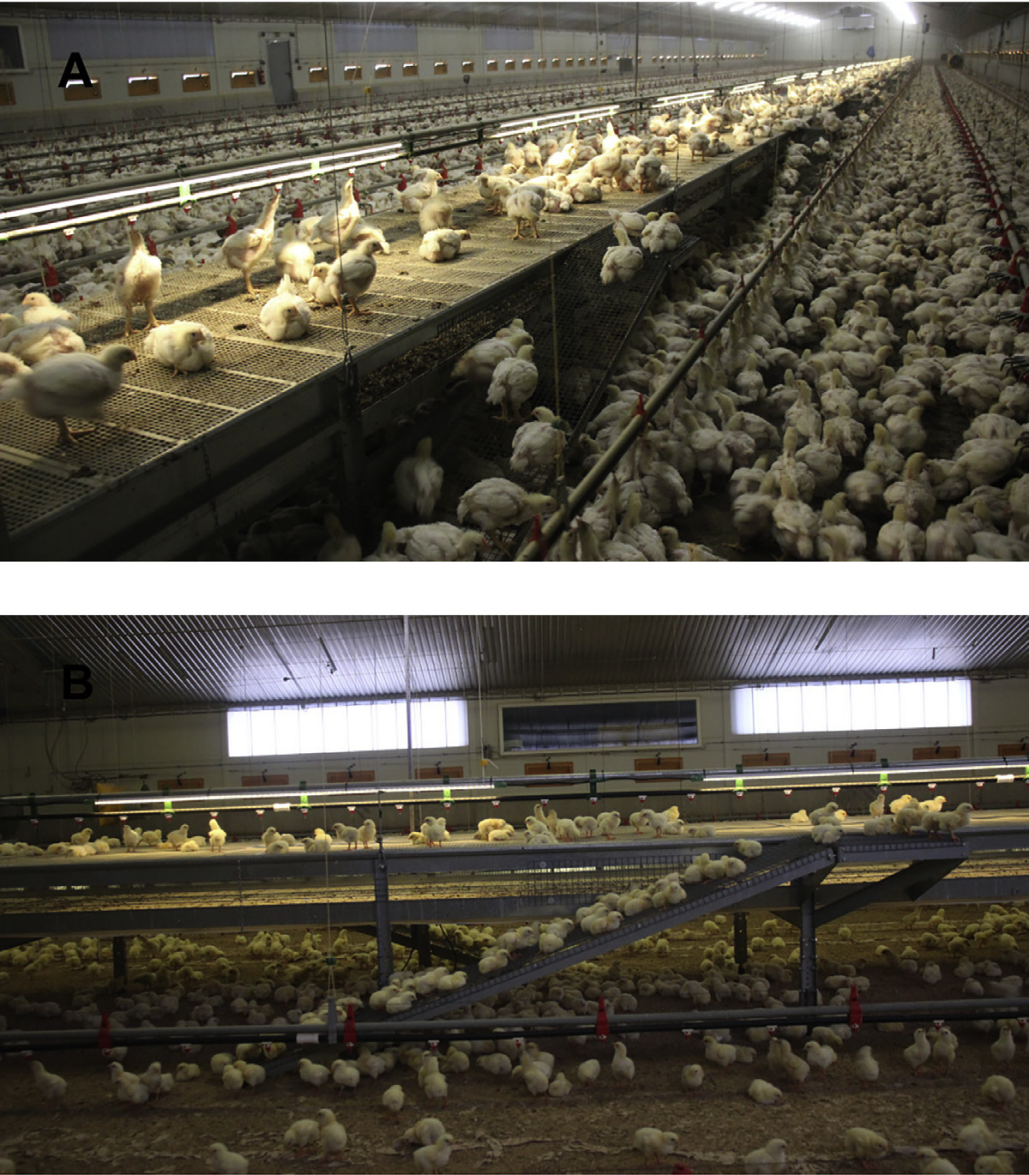
Elevated platform with perforated surface, manure belt, and ramps at d 21 (A) and d 3 (B) of the fattening period on farm A.

On 2 commercial farms in Germany, an elevated platform with manure belt (Fienhage Poultry-solutions GmbH, Lutten, Germany) was installed lengthwise in 1 barn (100 × 20 m (farm A) and 100 × 18 m (farm B)) on each farm (Figure 1A and B). The elevated platform extended the entire length of the barn (90 m) and was 1.2 m wide and 0.7 m high. To ease removal of the broiler chickens for slaughter, cleaning and disinfection of the barn the entire system could be lifted via a motor to the barn ceiling. The surface of the elevated platforms was made out of plastic elements measuring 0.75 m wide and 1.2 m long (mesh size 14 × 10 mm, bar width 5 mm), which are also used in cage systems for broiler production. The broilers could reach the elevated platforms via ramps (2.41 m long, 0.38 m wide) consisting of wire mesh (mesh size 19 × 19 mm). The upper part of each ramp served as a square transition area (0.41 × 0.41 m) between the ramp and surface of the platform. The angle between the ramp and barn floor was 20°. The ramps were installed alternately on both sides of the platform at a distance of 3.34 m apart. On the elevated platform an additional water line with drinking nipples (distance from nipple to nipple 0.17 m) and an LED line were installed.

The study included 3 replicates per farm with about 37,000 (farm A) and 33,000 (farm B) fast-growing broiler chickens (Ross 308, Emsland Brüterei (hatchery) GmbH, Dohren, Germany) raised in each barn until a slaughter age of 42 d and an average live weight of 2.9 kg. Thinning was performed at about d 33, when nearly 1/3 of the broilers were removed for slaughter. The average mortality in the flocks was 3.93% on farm A and 4.89% on farm B, which was similar to the mortality registered for previous flocks at the participating barns. All flocks were housed and managed under practical conditions in accordance with Council Directive 2007/43/EC (2023) (Council of the European Union, 2007) and the German Regulation on the Protection of Farm Animals and the Keeping of Production Animals (TierSchNutztV, 2021) as well as the requirements of the Animal Welfare Initiative for Poultry (Animal Welfare Initiative for Poultry, 2020). The maximum stocking density of 35 kg/m² according to the requirements of the Animal Welfare Initiative for Poultry was calculated on basis of the floor area of the barn including the area under the platform. The area on the perforated platform was not littered. Therefore, it was not counted as usable area in accordance with Council Directive 2007/43/EC (Council of the European Union, 2007) and provided about 108 m² additional space for the broilers (5–6% of the barn floor). The litter material consisted of straw granulates (about 750 g/m²) on farm A and spelt husk pellets (about 600 g/m²) on farm B. Standard broiler feed and water were provided ad libitum. Temperature, air humidity, and light program were regulated via a farm computer system in accordance with standard broiler regulations (Aviagen, 2018).

Farm A had permanent light during d 1 and 2. From the third day onward, there was a light period from 04:30 to 12:00 and from 13:00 to 22:30 with 15 min of dawn and 20 min of dusk periods each. The dawn periods were included in the light periods and the dusk periods in the dark periods. Farm B had a light period from 04:00 to 13:00 and from 15:00 to 00:00 from d 1 to 11. After d 11, the light periods lasted from 03:00 to 13:00 and from 14:00 to 00:00. Like farm A, farm B had 15 min of dawn and 20 min of dusk periods in its lighting program. The dawn and dusk periods were part of the light periods. For the lighting program, which differs from EU legislation, farm B had a special permit from the responsible veterinarian. An additional LED line was installed on the elevated platform, which could be controlled independently from the lighting program in the barn. Farm A used the LED line in addition to the barn light during the light periods, but it was turned off just before the barn light was. Farm B did not use the LED line. All chickens were vaccinated in the hatchery against infectious bronchitis (d 0). On the farms the birds were vaccinated against Gumboro (infectious bursal disease), Newcastle disease, and against infectious bronchitis for a second time.

### Behavioral Observations

**Figure 2:**
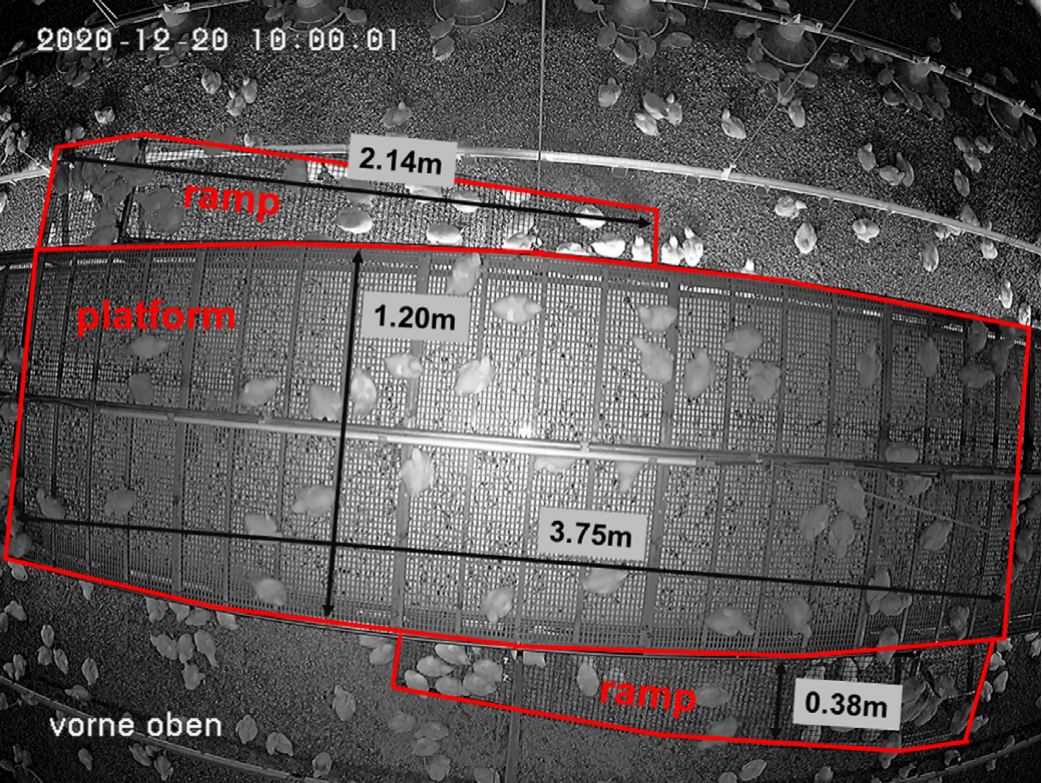
Camera view of the elevated platform and the ramps on farm B with the observed area outlined in red.

To evaluate the usage of the perforated platform, 3 fattening periods on each farm were videotaped. For this purpose, cameras with night vision (5MP Funk-Hybrid Set, Berghoch, Hartford Electronics GmbH, Dortmund, Germany) were installed on the perforated platform and videos were recorded (recorder B-HDDVR16DP, Berghoch, Hartford Electronics GmbH) on 2 d per wk for 24 h. The cameras were placed at a height of 2.5 m above 3 different areas of the platform in the front, the center, and the back of the barn. All areas filmed included the surface of the platform with the water and LED line and 2 opposing ramps (Figure 2). For video analysis the area on the platform was marked by a digital frame using the program GoldenRatio (Markus Welz, vs. 3.1.4, Krailling, Germany). The frame covered 4.5 m² of the platform surface and was 3.75 m long, which corresponded to 5 plastic slats and 1.2 m wide, which corresponded to the width of the platform. In addition, 2 ramps of 0.95 m² each were filmed (total filmed area on the ramps was 1.9 m²). During the observations, each animal was assigned to the location “platform” or “ramp.” The location “platform” corresponded to the area in the frame on the platform and the location “ramp” to the 2 ramps. To be assigned to 1 location, more than half of the broilers body had to be located in the area.

***Use of the Elevated Platform and Ramp (Scan Sampling).*** To evaluate the usage of the elevated perforated platform throughout the fattening period and during the light and dark periods, 1 d per wk was analyzed using scan sampling (d 4, 11, 18, 25, 32, 39). For the analysis, a screenshot was taken from the video material every 30 min during a 24-h period, for each of the 3 cameras (front, center, back of the barn). This resulted in *n* = 48 screenshots per day and camera, a total of 5,184 screenshots for the 6 sampling days. The number of broilers within the frame described above and on the ramps was determined by using the program ImageJ (Wayne Rasband, vs. 1.51q, National Institutes of Health, Bethesda, MD). For each screenshot, it was noted whether it was taken during a light or a dark period. Dark period was defined as the lights in the barn and on the platform were both turned off and the blinds closed, which usually corresponded to the dark period at night and the dark period at noon. It sometimes happened that the blinds were not closed in the dark period at noon, for example during repairs. Then the screenshots were recorded as light period, although they were taken during a dark period according to the lighting program. The scan sampling was performed by 1 observer. To calculate the intraobserver reliability, a subset of 40 scans at different ages and daytimes was analyzed again with an offset in time. One of the coauthors was trained to test for interobserver reliability and analyzed the 40 scans.

***Focal Animal Observation.*** To estimate the duration the broiler chickens stayed on the elevated platform, focal animals were observed. The footage from the camera in the center of the platform was chosen because the average number of broilers in this area did not differ between the farms. If the footage from the center camera was not usable due to technical issues, the footage from the camera at the front of the barn was used. This was the case on farm A for 1 observation time in the morning (4 focal animals in the middle of the fattening period) and for 2 observation times in the dark period (8 focal animals at the beginning of the fattening period). For the analysis, 3 d per fattening period were selected, hereafter referred to as “phases of the fattening period” (beginning: d 4, middle: d 18, and end: d 39). On each day a total of 12 broilers were observed continuously during 3 different time periods. The observation times were chosen for each farm adapted to the respective lighting program. During the dark period at night (1 h after the start of the dark period), 4 broilers were chosen for observation, 4 broilers in the light period in the morning (1 h after the start of the light period), and 4 broilers in the light period in the afternoon (1 h after the end of the dark period at noon). These times were chosen to take the different activity phases of the broiler chickens into account. In total, 212 focal animals were observed (beginning *n* = 68, middle *n* = 72, end *n* = 72). For the analysis of focal animal behavior, the focal animals observed during the 2 light periods were combined into 1 group observed during the light period (*n* = 144). Therefore, the group of birds observed in the dark period was smaller (*n* = 68). The focal animals were chosen pseudorandomly. The first animal that entered the platform from 1 of the ramps after the start time was chosen for observation. The video was rewound to the moment the chosen animal entered a ramp or was first visible on the ramp. From this time onward, the behavior and the location (platform or ramp) of the focal animal were observed. Thus, only broilers that entered the platform from 1 of the ramps were selected as focal animals. Broilers that only stayed on the ramps or entered the frame via the platform were not chosen as focal animals. The behavior patterns shown in Table 1 were differentiated and coded using the program INTERACT (Mangold International GmbH, vs. 17.1, Arnstorf, Germany). Only the behavior on the platform, not the ramps, was coded and analyzed. Observing broilers on the ramps was only necessary to choose focal animals. All behavior patterns were considered as mutually exclusive. The observation ended if a) the animal left the platform via one of the ramps, b) the animal left the platform or one of the ramps with a jump, c) the animal left the observation frame without leaving the platform or was not visible anymore, d) if 2 h had passed since the start of the observation (timeout). Referring to Norring et al. (2016), a change in the behavior pattern or the location was only coded if it lasted at least 3 s. To avoid observing 1 animal twice, the video was rewound after the end of the observation to the time point the focal animal entered the platform. The next focal animal that entered the platform from the other ramp was selected as the next focal animal. If no animal entered the platform from the other ramp within 10 min, the next animal observed on the same ramp was selected. At the observation time during the dark period, broilers did not move up the ramps. In this case, broilers were chosen from the platform by using a digital grid, again using the program Golden Ratio and a random number generator programmed with Microsoft Excel (2016). The intra- and interobserver reliability were calculated on the basis of the footage of 12 focal animals at different ages, as described for the scan sampling.

**Table 1:** Ethogram to record focal animal's behavior.

Behavior	Description
Locomotion	Animal is standing, walking or running. It can be engaged in other activities like preening and pecking.
Sitting	Animal is lying with a leg to the side or sitting with the legs under the body. It can be engaged in other activities like preening, pecking or resting.^1^
Drinking	Animal is pecking at the drinking nipples.^2^

1 According to Forslind et al. (2021).

2 According to de Jong and Gunnink (2019).

### Statistical Analysis

Statistical analysis was performed using the SAS software (Statistical Analysis Institute, vs. 9.4, Cary, NC). The intra- and interobserver reliability were calculated using Krippendorff's alpha (Hayes and Krippendorff, 2007). The number of bootstraps was set to 2,000; the data type was set to metric for each parameter. For the scan sampling, the observer reliability was calculated separately for the number of broilers on the platform and the ramps. The observer reliability for the focal animals was calculated for all behavior patterns together. The categorization was done according to the suggestion of Landis and Koch (1977) (<0.00 = poor, 0.00–0.20 = slight, 0.21–0.40 = fair, 0.41–0.60 = moderate, 0.61–0.8 = substantial, 0.81–1.00 = almost perfect). Means and standard deviations (**SD**) were calculated for descriptive statistics for all parameters.

For the scan sampling, 2 generalized linear mixed models were calculated to analyze 1) the effect of the week of life (1–6), location (platform, ramp) and their interactions on the number of broilers per m² in the observed areas (platform and ramp), and 2) the effect of the week of life (1–6) and lighting period (light, dark) and their interactions on the number of broilers per m² only on the platform (without ramp). The hierarchical structure of the experimental design was taken into account in the random statement. Therefore for (1) replicate was nested in farm, week of life in replicate, camera position in week of life, time per camera position and time in location. For (2), replicate was nested in farm, week of life in replicate, camera in week of life and time in camera and lighting period respectively. Pairwise comparisons were made using Tukey-Kramer tests. The level of significance was set to *P* < 0.05. Upper and lower limits of the 95% confidence interval are given in brackets after the *P* value.

For focal animal observations, only the behavior recorded on the platform (not on the ramps) was included in the statistical analysis. Again, generalized linear mixed models were used to evaluate the effect of the fixed factors farm (A, B), lighting phase (light, dark) and phase of the fattening period (beginning, middle, end) and their respective interactions on the respective target variable. These included the total duration spent on the platform (“total”) [hh:mm:ss] and the duration [hh:mm:ss] as well as the frequency of behavioral events of the behavior patterns “Locomotion,” “Sitting,” and “Drinking” [bout per min]. In addition, the mean and the maximum duration of the Sitting bouts were calculated for each focal animal. The effect of the above mentioned fixed factors and the interaction between both on the mean and maximum duration of the Sitting bouts were analyzed. All parameters were analyzed separately. Again, pairwise comparisons were made using Tukey-Kramer tests and the level of significance was set to *P* < 0.05. Upper and lower limits of the 95% confidence interval are given in brackets after the *P* value. Broilers showed Drinking only during the light period. Therefore, only descriptive statistics were performed.

## RESULTS

The intra- and interobserver reliabilities were “almost perfect” with values ranging from 0.82 to 1.00 for the scan sampling and the focal animal observation. The values of Krippendorff's alpha coefficients are presented in Table 2.

**Table 2:** Observer reliabilities for scan sampling (*n* = 40 scans) and the focal animal observation (*n* = 12 focal animals).

Method	Location	Krippendorff's alpha Intraobserver	Krippendorff's alpha Interobserver
Scan sampling	Platform	1.00	1.00
	Ramp	0.97	0.96
Focal animal observation	Platform	0.83	0.82

### Use of the Elevated Platform and the Ramp

**Figure 3:**
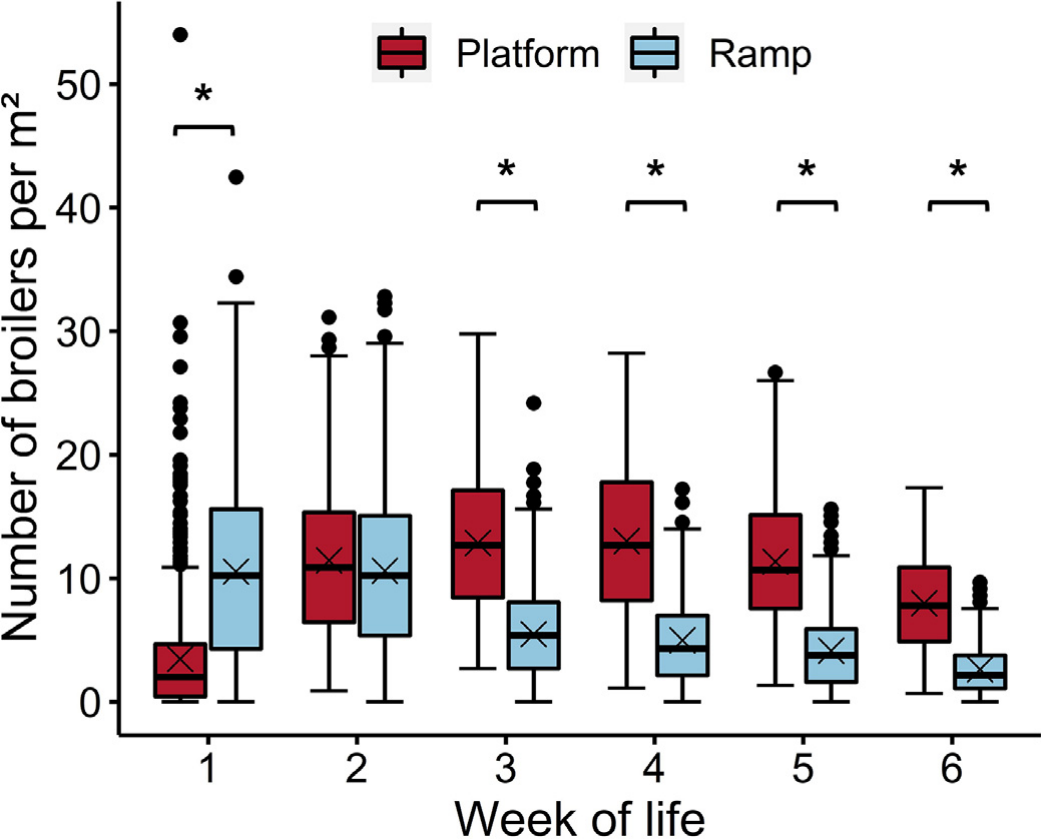
Number of broilers per m² depending on the week of life and the location in the observed area. Results are presented as boxplots (data range, median, and lower and upper quartile; outliers are shown in the graph as dots and means as crosses). Differences at *P* < 0.05 between the platform and ramp within the week of life are marked by asterisks.

Averaged over the entire fattening period, the number of broilers per m² was 9.92 (SD 6.23) on the platform and 6.47 (SD 5.79) on the ramp. An interaction effect between the week of life and the location (platform or ramp) was found (*F*_(5, 9683)_ = 851.74, *P* < 0.001), as shown in Figure 3. In wk 1, more broilers per m² were found on the ramp (10.55, SD 7.27) than on the platform (3.48, SD 4.52) (*t* = |−15.80|, *P* < 0.001, [−8.55, −5.62]). In contrast, from wk 3 until the end of the fattening period, the number of broilers per m² was higher on the platform (on average 11.30, SD 5.17) than on the ramp (on average 4.31, SD 3.12) (all *t* > |11.95|, all *P* < 0.001). Platform use increased during the fattening period up to wk 4 (13.00, SD 6.29), with more broilers per m² on the platform in wk 2 to 5 than in wk 1 (wk 2: *t* = |−4.00|, *P* = 0.004, [−14.51, −1.45]; wk 3: *t* = |−4.77|, *P* < 0.001, [−16.07, −3.00]; wk 4: *t* = |−4.80|, *P* < 0.001, [−16.11, −3.05]; wk 5: *t* = |−3.96|, *P* = 0.004, [−14.45, −1.39]). Toward the end of the fattening period, the recorded broilers per m² on the platform decreased again to 7.98 (SD 3.71) in wk 6. In contrast, the use of ramps reached its peak at the beginning of the fattening period and decreased from wk 2 onward. This was reflected by a higher number of broilers per m² on the ramp in wk 1 and 2 (on average 10.58, SD 7.04) compared to wk 6 (2.63, SD 2.16) (all *t* > |3.97|, all *P* = 0.004, wk 1 [1.40, 14.45], wk 2 [1.45, 14.51]).

**Figure 4:**
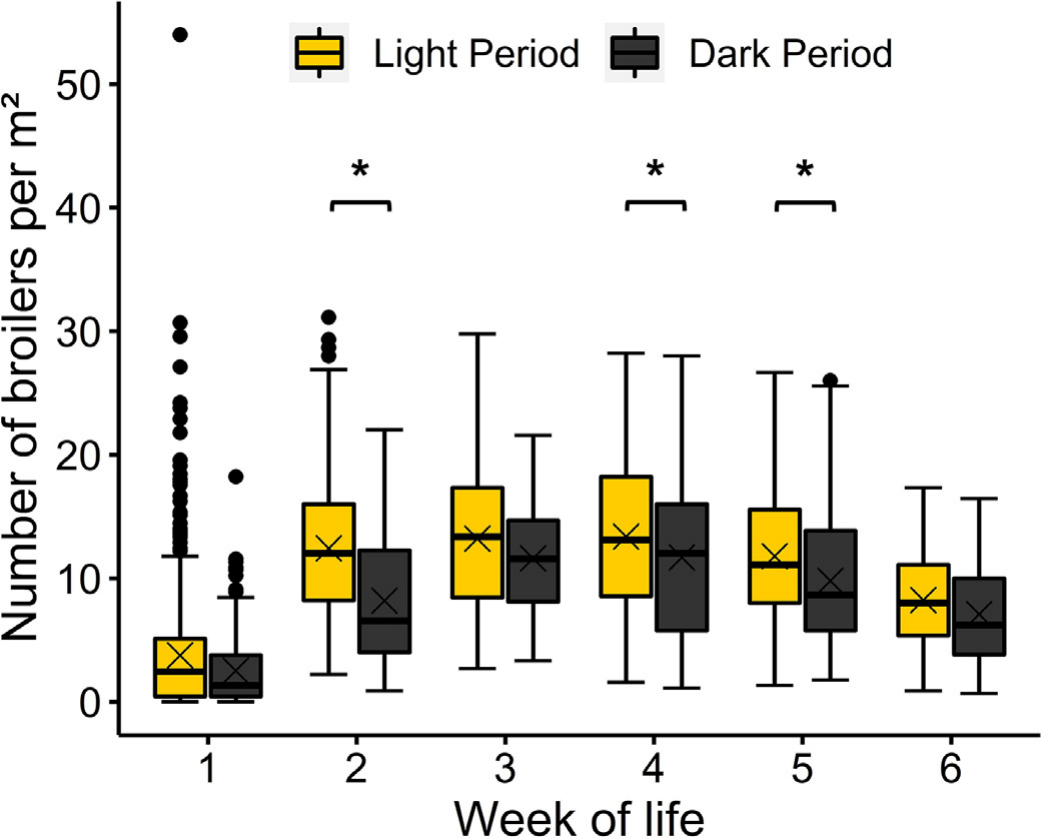
Number of broilers per m² on the platform depending on the lighting phase and the week of life. Results are presented as boxplots (data range, median, and lower and upper quartile; outliers are shown in the graph as dots and means as crosses). Differences at *P* < 0.05 between dark and light periods within the week of life are marked by asterisks.

On average, 10.39 (SD 6.24) broilers per m² were counted on the platform during the light periods and 8.32 (SD 5.90) during the dark periods. There was an interaction between the week of life and the lighting phase (*F*_(5, 4758)_ = 13.21, *P* < 0.001). In wk 2, 4, and 5, pairwise comparisons showed a higher number of broilers per m² during the light periods than during the dark periods (wk 2 and 4: *t* = |−3.33|, *P* = 0.042, [−3.10, −0.03]); wk 5: *t* = |−3.07|, *P* = 0.004, [−3.45, −0.33]). This was not the case in wk 1, 3, and 6 (Figure 4). During the light periods as well as during the dark periods, the number of broilers per m² on the platform was higher in wk 3 and 4 than in wk 1 (light, wk 3: *t* = |−3.64|, *P* = 0.015, [−18.31, −0.97]; light, wk 4: *t* = |−3.67|, *P* = 0.013, [−18.39, −1.05]; dark, wk 3: *t* = |−3.47|, *P* = 0.026, [−18.05, −0.54]; dark, wk 4: *t* = |−3.43|, *P* = 0.030, [−17.93, −0.43]). Additionally, during the light periods, the number of broilers per m² was higher in wk 2 than in wk 1 (*t* = |−3.28|, *P* = 0.048, [−17.38, −0.04]).

### Focal Animal Observation

***Total Duration on the Platform.*** On average, focal animals stayed in the observation frame (4.5 m²) for 00:27:22, SD 00:43:17 [hh:mm:ss]. The total duration depended on the light period (*F*_(1, 202)_ = 692.30, *P* < 0.001) and the phase of the fattening period (*F*_(2, 202)_ = 9.56, *P* < 0.001). With an average duration time of 01:54:23, SD 00:21:17 [hh:mm:ss] during the dark period, broilers spent more time on the platform than those broilers observed during the light period (00:19:54, SD 00:27:21 [hh:mm:ss]) (*t* = |26.31|, *P* < 0.001, [5207.87, 6051.66]). The broilers spent more time on the platform in the middle (00:54:47, SD 00:50:45 [hh:mm:ss]) and at the end (00:59:22, SD 00:50:33 [hh:mm:ss]) of the fattening period than at the beginning (00:35:41, SD 00:49:23 [hh:mm:ss]) (middle: *t* = |−3.48|, *P* = 0.002, [−1550.15, −298.02]; end: *t* = |−4.10|, *P* < 0.001, [−1712.15, −460.02].

**Figure 5:**
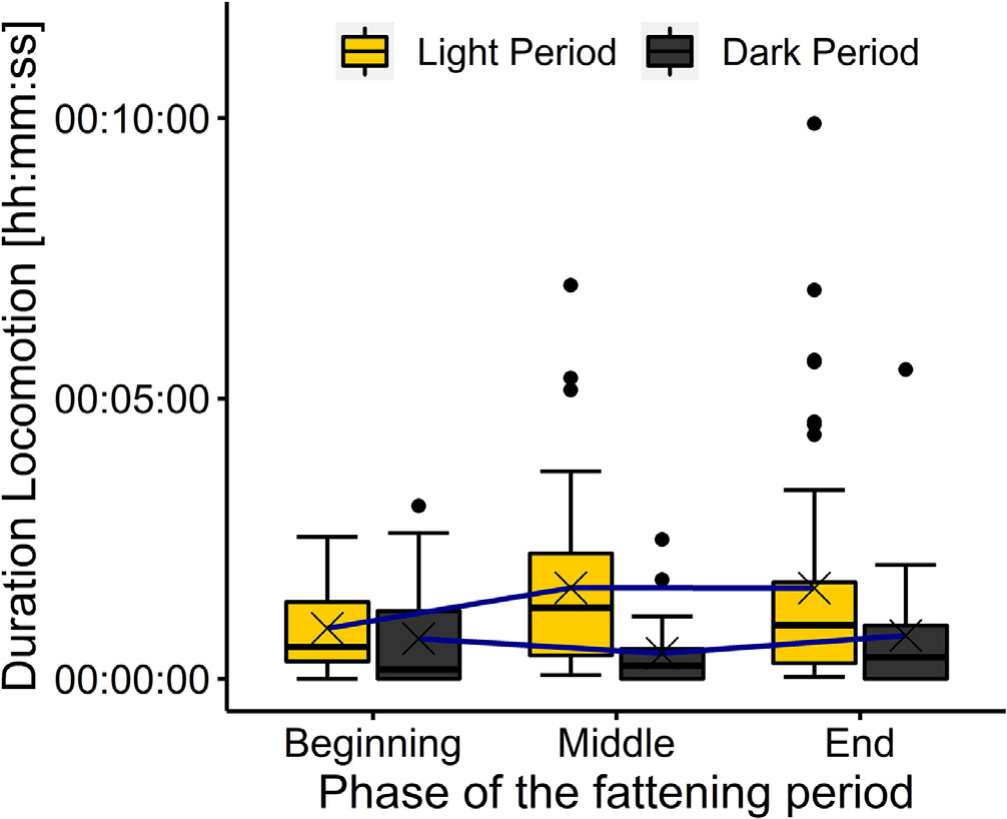
Duration of Locomotion in the different phases of the fattening period and lighting phases. Results are presented as boxplots (data range, median, and lower and upper quartile; outliers are shown in the graph as dots and means as crosses). Differences at *P* < 0.05 are marked by asterisks.

***Locomotion.*** Broilers showed Locomotion for a mean duration of 00:01:21, SD 00:03:18 [hh:mm:ss] at a frequency of 0.24, SD 0.33 bouts per min, which corresponds to 15.88% of the total time broilers spent in the observed area on the platform. Of the broilers observed during the light period, 97.92% showed Locomotion, while 67.65% showed Locomotion during the dark period. In all phases of the fattening period, broilers spent only a small amount of time with Locomotion (beginning 00:01:29, SD 00:05:21 [hh:mm:ss]; middle: 00:01:14, SD 00:01:24 [hh:mm:ss]; end: 00:01:20, SD 00:01:52 [hh:mm:ss]). The duration of Locomotion in the different phases of the fattening period and lighting phases is presented in Figure 5. There was an interaction effect of the lighting phase and phase of the fattening period for the duration of Locomotion (*F*_(2, 202)_ = 3.53, *P* = 0.031). However, no differences could be found in the pairwise comparisons to specify this.

Broilers showed Locomotion with a frequency of 0.34, SD 0.35 bouts per min during the light period and with 0.03, SD 0.07 bouts per min during the dark period. The lighting phase in interaction with the farm was found to affect the frequency of Locomotion (*F*_(1, 202)_ = 4.04, *P* = 0.046), with broilers on farm A showing Locomotion in a higher frequency (0.46 bouts per min, SD 0.47) than broilers on farm B (0.36 bouts per min, SD 0.46) during the light period (*t* = |−3.27|, *P* < 0.01). The phase of the fattening period had no effect on the frequency of Locomotion.

***Sitting.*** On average, the focal animals showed Sitting behavior for 00:48:24, SD 00:50:50 [hh:mm:ss], which equates to a mean of 80.60% of the observation time. Of the broilers observed during the light period, 90.28% showed Sitting behavior, while all broilers showed Sitting behavior during the dark period. For the duration of Sitting, an effect of the lighting phase was found (*F*_(1, 202)_ = 769.28, *P* < 0.001). With an average of 01:53:05, SD 00:22:01 [hh:mm:ss], the broilers spent more time Sitting during the dark period than during the light period (00:17:51, SD 00:25:37 [hh:mm:ss]) (*t* = |27.74|, *P* < 0.001). Broilers observed during the dark period spent an average of 98.55% of the observation time Sitting, while those observed during the light period spent an average of 72.12% of the observation time Sitting. The phase of the fattening period had an effect on the duration of Sitting (*F*_(2, 202)_ = 10.83, *P* < 0.001). Broilers spent more time Sitting in the middle (00:53:03, SD 00:51:01 [hh:mm:ss]) and at the end (00:57:20, SD 00:50:17 [hh:mm:ss]) of the fattening period than at the beginning (00:34:00, SD 00:48:47 [hh:mm:ss]) (middle: *t* = |−3.76|, *P* < 0.001, [−1552.59, −355.35]; end: *t* = |−4.33|, *P* < 0.001, [−1695.91, −498.68]).

**Figure 6:**
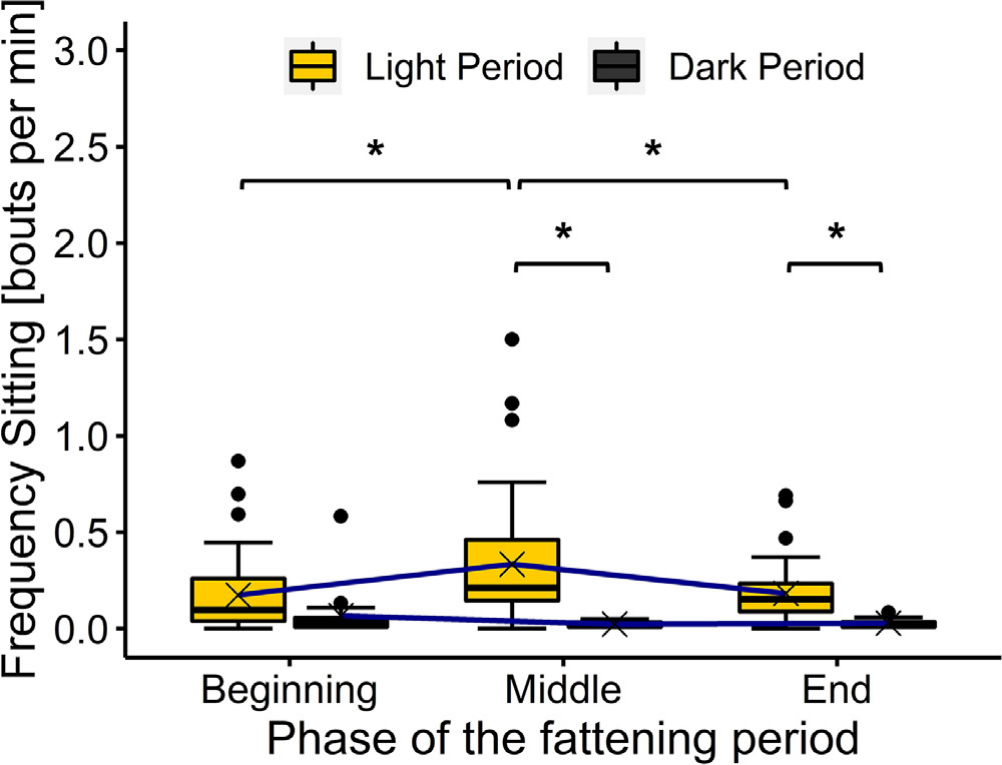
Frequency of Sitting during the different phases of the fattening period and lighting phases. Results are presented as boxplots (data range, median, and lower and upper quartile; outliers are shown in the graph as dots and means as crosses). Differences at *P* < 0.05 are marked by asterisks.

The frequency of Sitting was on average 0.23 (SD 0.24) bouts per min in the light period and 0.04 (SD 0.07) bouts per min in the dark period. An interaction effect for the lighting phase and phase of the fattening period was found (*F*_(2, 202)_ = 4.94, *P* = 0.008) (Figure 6). Pairwise comparisons revealed a higher frequency of Sitting during the light period than during the dark period in the middle and the end of the fattening period (middle: light: 0.33 (SD 0.31), dark: 0.02 (SD 0.02) (*t* = |−5.24|, *P* < 0.001, [−0.41, −0.12]); end: light: 0.18 (SD 0.15), dark: 0.03 (SD 0.02) (*t* = |−3.08|, *P* = 0.002, [−0.28, −0.01])). During the light period, the highest frequency of 0.33, SD 0.31 bouts per min was found in the middle of the fattening period. At the beginning and at the end of the fattening period, the frequencies were lower (beginning: 0.17, SD 0.19 (*t* = |−4.15|, *P* = 0.001, [−0.27, −0.05]); end: 0.18, SD 0.15 (*t* = |−3.90|, *P* = 0.002, [−0.26, −0.04])). For the broilers observed during the dark period, no difference between the different phases of the fattening periods was found.

The lighting phase had an effect on the duration of the maximum Sitting bouts per focal animal (*F*_(1, 202)_ = 643.68, *P* < 0.001). Specifically, the duration of maximum Sitting bouts was longer during the dark period (01:23:57, SD 00:33:38 [hh:mm:ss]) than during the light period (00:08:44, SD 00:10:07 [hh:mm:ss]) (*t* = |25.37|, *P* < 0.001, [41134.66, 4831.50]). The duration of maximum Sitting bouts differed between the phases of the fattening period (*F*_(1, 202)_ = 8.70, *P* < 0.001), with longer maximum Sitting bouts in the middle (00:38:14, SD 00:43:58 [hh:mm:ss]) and at the end of the fattening period (00:36:31, SD 00:38:15 [hh:mm:ss]) than at the beginning (00:23:19, SD 00:38:53 [hh:mm:ss]) (middle: *t* = |−4.06|, *P* = 0.01, [−1405.98, −371.92], end: *t* = |−2.98|, *P* = 0.001, [−1170.07, −136.02]). Furthermore, the farm was found to have an effect on the maximum Sitting bouts (*F*_(1, 202)_ = 4.04, *P* = 0.046), with farm B showing longer maximum Sitting bouts (00:34:49, SD 00:42:10 [hh:mm:ss]) compared to farm A (00:30:59, SD 00:39:36 [hh:mm:ss]) (*t* = |2.01|, *P* = 0.046, [6.84, 703.68]).

The duration of the mean Sitting bouts per focal animal observed during the light period was 00:03:29, SD 00:04:02 [hh:mm:ss] and 00:59:42, SD 00:43:20 [hh:mm:ss] during the dark period. An interaction effect between farm and lighting phase was found (*F*_(1, 202)_ = 4.47, *P* < 0.358). The duration of mean Sitting bouts was longer during the dark period then during the light period for both farms (farm A: light 00:02:50, SD 00:03:36; dark 00:51:30, SD 00:40:28 [hh:mm:ss] (*t* = |9.76|, *P* < 0.001, [2144.41, 3694.14]) and farm B: light 00:04:08, SD 00:04:21; dark 01:08:56, SD 00:45:12 [hh:mm:ss] (*t* = |12.23|, *P* < 0.001, [3022.65, 4648.01])). However, broilers on farm A showed shorter mean Sitting bouts during the dark period (00:51:30, SD 00:40:28 [hh:mm:ss]) compared to farm B (01:08:56, SD 00:45:12 [hh:mm:ss]) (*t* = |2.78|, *P* < 0.030, [66.303, 1921.66]). The phase of the fattening period did not affect the mean duration of Sitting bouts per broiler.

***Drinking.*** Of the broilers observed during the light period, 39.58% showed Drinking behavior, while only 1 bird drank during the dark period. Therefore, the dark period was excluded from the analysis for Drinking. On average, focal animals showed Drinking behavior for a duration of 00:00:40, SD 00:01:30 [hh:mm:ss], representing 3.21% of the total time spent on the platform. The average frequency of Drinking was 0.07, SD 0.18 bouts per min.

***Percentages of the Different Ways in Which Focal Animals Left the Observation Frame.*** The percentages of the different ways in which focal animals left the observation frame are presented in Table 3. Most focal animals (57.55%) left the observation frame without leaving the platform. In addition, this was the most common way of observation completion of all broilers observed during the light period (81.3%). The observation of 32.08% of the focal animals was stopped because the maximum observation time of 2 h was reached. With 91.2% of the broilers observed during the dark period, this was the most frequent way the observation ended for the dark period. In total, 6.13% of all observed focal animals (light and dark period) left the platform walking down a ramp and 4.25% jumped off the platform or a ramp. Jumping off the platform occurred most often at the beginning of the fattening period (10.3% of focal animals observed at the beginning of the fattening period), whereas in the middle of the fattening period, no animal, and at the end, 2.8% of the observed broilers jumped off the platform.

**Table 3:** Percentages of the different ways in which focal animals left the observation frame during the light and dark period and during the different phases of the fattening period.

Ways in which focal animals left the observation frame	Lighting phase		Phase of the fattening period			Total % (*n* = 212)
	Light (*n* = 117)	Dark (*n* = 68)	Beginning (*n* = 68)	Middle (*n* = 72)	End (*n* = 72)	
Frame	81.3%	7.4%	58.8%	56.9%	56.9%	57.5%
Timeout	4.2%	91.2%	23.5%	36.1%	36.1%	32.1%
Ramp	9.0%	0.0%	7.4%	6.9%	4.2%	6.1%
Jump	5.6%	1.5%	10.3%	0.0%	2.8%	4.2%

## DISCUSSION

The purpose of this study was to evaluate the use of an elevated platform with a perforated surface and manure belt by fast-growing broilers. The study assessed under commercial conditions how broilers use the platform and ramps at different ages and whether the broilers use the platform to sit elevated during the dark period. Furthermore, it was analyzed how long focal animals stayed on the platform and their behavior was described in more detail.

The broilers occupied the elevated platform and ramps throughout the entire fattening period. The platform used in this study, with a height of 70 cm, was relatively high compared to other studies (Norring et al., 2016; Bach et al., 2019; Malchow et al., 2019; Baxter et al., 2020). This dimension had to be chosen to enable daily monitoring of the broilers under the platform. Nevertheless, the average numbers of 10.39 broilers per m² in the light period and 8.32 broilers per m² in the dark period were only slightly lower than in previous studies. Norring et al. (2016) counted 15 broilers per m² on a 30 cm high platform with ramps at daytime and about 9.8 broilers per m² at nighttime. Baxter et al. (2020) found 11.5 broilers per m² at daytime on suspended platforms made of plastic mesh without ramps but adjusted to the growth of the broilers. The number of broilers per m² on the ramp in the current study was on average 6.47. Those results suggest that the design of the platform and the ramps was suitable for the fast-growing broiler chickens and their limited physical abilities. The combination of a broad ramp (38 cm), where 2 broiler chickens could pass each other, a flat inclination angle of 20°, and a connecting element between the ramps and the platform could be the key for these results. The importance of ramp design was also mentioned in Malchow and Schrader (2021), suggesting an inclination angle of 29.1° and a 60 cm wide ramp as suitable for fast-growing broilers.

In the course of the fattening period, platform use increased in the first 4 to 5 wk, as reported by several studies (Bailie et al., 2018; Malchow et al., 2018; Schomburg et al., 2022). During wk 1, few chicks entered the platform (3.48 broilers per m²), whereas the highest amount of broilers was counted on the ramp (10.55 broilers per m²). A possible explanation is that the chicks first explored the ramps and afterward started to use the platform. This could be comparable to pullets spending time under perches before the first perching attempts (Heikkilä et al., 2006). Another reason could be that the mesh size of the grid of the ramps was not ideal for up to 1-wk-old chicks, proving too uncomfortable to walk up the whole ramp. Nevertheless, in wk 2 the same number of broilers was found on the ramp and the platform, and from wk 3 onward more broilers per m² were found on the platform than on the ramp. These results are in accordance with Bailie et al. (2018), who found that broilers preferred to perch on suspended platforms rather than bars or ramps. With 13.00 broilers per m², the highest number of broilers on the platform was found in wk 4, which was also reported by previous studies (Norring et al., 2016; Bailie et al., 2018; Malchow and Schrader, 2021). Toward the end of the fattening period in wk 6, the number of broilers on the platform decreased to 7.98 per m². Norring et al. (2016) and Bailie et al. (2018) also noted a lower number of broilers on elevated platforms at the end of the fattening period and attributed this to increasing space requirements and decreasing animal activity. The higher weight and therefore leg weakness could also contribute to lower platform use (Bessei, 2006). Further research is required to identify the reasons for lower platform use at the end of the fattening period. Nevertheless, the result should be considered when platforms are installed to provide additional space.

Comparing light and dark periods, the expectation that broilers would increasingly use the platform for elevated resting during the dark periods (McBride et al., 1969; Newberry et al., 2001) were not confirmed by the number of birds on the platform. Instead, platform use in wk 2, 4, and 5 was higher during the light periods than during the dark periods. Malchow et al. (2018) and Norring et al. (2016) also found that elevated structures were used less by broiler chickens during the dark periods than during light periods. A possible explanation for this could be that the fast-growing broilers are too young to perch. Chicks are brooded by the hen on the ground until the hen begins to take them to lower branches at night at 6 wk of age (McBride et al., 1969). Heikkilä et al. (2006) also found that layer chicks at an age of 6 wk only spent 5.6% of their nighttime observations on perches. Fast-growing broilers are already heavy and inactive by this time and may never develop nighttime roosting behavior to the extent laying hens do. This theory is supported by another study by Malchow and Schrader (2021) comparing platform use between fast-growing broilers and dual-purpose chickens. Fast-growing broiler chickens used the platform to a comparable degree between light and dark periods. Dual-purpose chickens used the platform more often during the light periods up until wk 7 of the fattening period; afterward, the usage was higher during dark periods. However, results contradict those of Riber et al. (2007), who found laying hens starting to use perches during nighttime already at an age of 20 d. Using the LED line to attract the birds to move up the platform for nighttime roosting, as it is done in multitier systems for laying hens, would have been an option. In the present study, this was not applied, as this was assumed to negatively influence the distribution of broilers in the barn. Another explanation for a higher platform use during the light periods might be a higher animal activity during light periods, which could result in more broilers entering the platform than during the dark periods. This was supported by our results showing that during the light period, broilers were more active and likely to leave the observation frame while still staying on the platform. Another factor may be changing climatic conditions on the platform at night. At the beginning of the fattening period, chicks have a high need for heat (Aviagen, 2018). Broilers on the platform are cooled by air movement underneath the perforated surface. In addition, birds sitting at the edge of the platform do not have a neighbor on 1 side. Even without underfloor heating, it might be warmer for chicks in the litter on the ground of the barn than on the elevated platform, especially at night. On the other hand, due to their high production of metabolic heat (Tickle et al., 2018), older broilers could be attracted by a cooler area on the platform. This could be the case especially during daytime when it is warmer in the barn. Further studies with permanent collection of climate data on elevated platforms are needed to evaluate the influence of temperature on the usage of platforms. In contrast to Malchow et al. (2018) and Norring et al. (2016), a study by Schomburg et al. (2022) found a higher usage at nighttime than at daytime of a 50 cm high platform from wk 3 of life onward. This deviating result could be due to the fact that Schomburg et al. (2022) used a different method. Instead of video analysis by scan sampling, they used a weighing system and developed an algorithm to continuously evaluate the usage by fast-growing broilers.

Even if the results could not show a higher frequency of using the platform during the dark period compared to the light period, the observation of focal animals revealed differences in the behavior. The broilers stayed longer on the platform during the dark period than during the light period. This may be explained by the fact that during the dark period broilers were mostly Sitting and therefore did not leave the platform or the observation frame. This is also reflected in the result that 91.2% of the broilers observed during the dark period exceeded the maximum observation time of 2 h. During the light period, broilers were more active and were likely to leave the observation frame but to stay on the platform (81.3% of the broilers observed during the light period). In both cases, the time broilers spent on the platform evaluated in the current study might be underrated. Considering that broilers visit the feeders an average of 4 times per h (Lewis and Hurnik, 1990), another explanation for the shorter total time broilers stayed on the platform during the light period might be that they had to leave the platform to feed. Baxter et al. (2020) found a shorter mean perching duration of 00:05:52 [hh:mm:ss] during the day on suspended platforms, which could be explained by the shorter maximum observation time of 00:30:00 [hh:mm:ss] in that study. In the current study, broilers stayed longer on the platform in the middle and at the end of the fattening period than at the beginning. This finding is consistent with that of Baxter et al. (2020), who found that the perching time on suspended platforms increased with age. The fact that broiler chickens at the end of the fattening period are less active due to high body weight and associated leg pain (Bessei, 1992; Weeks et al., 2000; Bizeray et al., 2002; Norring et al., 2016) might have contributed to this result. Younger broilers are more active and it is more likely that they leave the observation frame or the platform. To evaluate the real time broilers spend on the platform, further studies could use a tracking system to track individual broilers from the point they enter a ramp. In general, the results of the focal animal observation should be interpreted with caution due to the relatively high standard deviations. This is likely to be related to the highly differing total duration focal animals were observed for. Consequently, the durations of the observed behavior patterns of focal animals differed. In addition, the number of focal animals observed in the current study was limited due to long video analysis times. A tracking system or alternative methods of behavioral analysis such as those used by Schomburg et al. (2022) may be appropriate to reduce analysis time and increase the sample size at the same time.

As hypothesized, both broilers observed during the light period and the dark period spent a large amount of their time on the platform Sitting (80.6%) and a lower proportion showed Locomotion (15.9%). Due to the angle of the camera, it was not possible to determine whether the broilers were awake or asleep. Therefore, Sitting also included broilers preening or pecking at the ground. Nevertheless, the high proportion of broilers Sitting may indicate that they used the platform also for resting. As with the total time spent on the platform, the duration of Sitting was longer in the middle and at the end of the fattening period than at the beginning, which is in accordance with Weeks et al. (2000) and Bizeray et al. (2002) who found an increase in lying behavior with age.

When interpreting the results of the duration of Locomotion, it should be kept in mind that active broilers were more likely to leave the frame and were not observed anymore. Therefore, the duration of Locomotion could be underrated. In the current study, broilers showed Locomotion in a comparable frequency to the frequency of Sitting. This finding indicates a frequent change between Sitting and Locomotion most of the time.

Broiler behavior is strongly related to light intensity (Newberry et al., 1988; Alvino et al., 2009; Schwean-Lardner et al., 2014). In accordance with the results of Norring et al. (2016), the current study found that broilers observed during the dark period were mostly inactive and showed Sitting less frequently than the broilers during the light period. Prior studies have stated the importance of undisturbed rest for health, welfare, and performance of broiler chickens and supposed that resting behavior is often disturbed by active broilers (Schwean-Lardner et al., 2014; Yngvesson et al., 2018; Forslind et al., 2021). Elevated structures are supposed to reduce disturbances (Yngvesson et al., 2018; Forslind et al., 2021). In the current study, the mean duration of the maximum Sitting bouts was longer during the dark period, which might be due to the higher motivation to rest during night than during the daytime. During the light period, broilers entering the platform or searching for the water line might have disturbed broilers Sitting on the platform. Furthermore, groups of broilers were running along the platform and were likely to disturb Sitting broilers. The platform in the current study was equipped with a water line and an LED line. It seems possible that this encouraged active behavior on the platform and may have increased disturbances, which is contrary to the aim of providing a resting area on the platform. Presumably, the fact that only farm A used the LED light influenced the broilers’ behavior, as the frequency of Locomotion was higher and the mean and maximum Sitting bouts were shorter on farm A compared to farm B. On the other hand, no difference was found between the farms for the duration of Sitting, Locomotion and the total time spent on the platform. It is likely that these parameters would also be affected, if the broilers at farm A perceived the platform as an area of active behavior to a greater extent than the broilers on farm B. In any case, differences between the light programs on the farms, such as the different total duration of the dark periods, might have affected the results of the study. An implication of those findings is that disturbances might be reduced by optimizing the design of the elevated structure. Shorter platforms, a waiver of the water line, wider transition space, or lower stocking densities might decrease the frequency of disturbances.

The Drinking frequency is slightly higher than the frequency found by Lewis and Hurnik (1990) in about 5-wk-old broilers. Weeks et al. (2000) found that chickens spent 3% of the day Drinking, which is in accordance with the results in the current study. Although the water line was used by the broiler chickens, it does not answer the question whether a water line on a platform is necessary. With regard to the aim of giving broilers the opportunity to rest undisturbed, the water line could be counterproductive.

In conclusion, the fast-growing broilers used the elevated platform with perforated surface during the entire fattening period. The broilers visited the platform mainly during the light period and showed to a high proportion inactive behavior. The material and structure of the platform appear to be suitable for all ages considered here. The study implies that fast-growing broiler chickens use elevated structures if they are adjusted to their needs. The elevated platform is an option to structure broiler barns. It provides additional space and gives the broilers the opportunity to sit elevated.
